# Penetrating injuries of the posterior dural venous sinuses: a systematic review of injury mechanisms and repair techniques

**DOI:** 10.3389/fneur.2026.1827710

**Published:** 2026-07-29

**Authors:** Kashif Qureshi, Kivanc Yangi, Egemen Gok, Jack T. Olson, Jarett E. Prince, Michell Goyal, Mark C. Preul

**Affiliations:** The Loyal and Edith Davis Neurosurgical Research Laboratory, Department of Neurosurgery, Barrow Neurological Institute, St. Joseph's Hospital and Medical Center, Phoenix, AZ, United States

**Keywords:** confluence of sinuses, cranial sinuses, head injuries, neurosurgical procedures, penetrating, sigmoid sinuses, transverse sinuses, vascular system injuries

## Abstract

Penetrating injuries to the posterior dural venous sinuses (DVSs), which include the transverse sinus, sigmoid sinus, and torcular Herophili, are rare, life-threatening events with no standardized management protocols. This study aims to elucidate the injury mechanisms, management strategies, and outcomes associated with these injuries. A systematic review of all reported cases of penetrating posterior DVS injury was conducted on February 08, 2026. The systematic review yielded 10 studies comprising 43 patients. Demographic data were reported for eight of these 43 patients (18.6%). Seven (87.5%) of these patients were male, and one (12.5%) was female. Mean (SD) patient age was 25.8 (16.6) years (range, 5–52 years). The most common mechanism of injury was penetrating injury (38 of 43, 88.4%). The transverse sinus was the most frequently injured sinus (83.7%); reported management techniques for transverse sinus injuries included suture repair, grafting, and packing. Sigmoid sinus injuries were identified in five of 43 patients (11.6%); most of these injuries were managed with tamponade, but one patient (20%) died. Torcular Herophili injuries were rare (2 of 43 patients, 4.7%) and often hemodynamically unstable. Direct repair (e.g., suture, patch, or graft) was associated with improved survival. These reports showed that management of penetrating posterior DVS injuries may be tailored to the sinus involved, and evidence supports reconstructive strategies to preserve venous outflow whenever possible. Although transverse sinus ligation may be tolerated in cases of contralateral dominance, sigmoid sinus injuries are associated with poorer outcomes and were typically managed with tamponade. In contrast, patients with torcular Herophili injuries were shown to have favorable outcomes when repaired to preserve flow. This integrated review provides a foundational framework for managing complex venous sinus injuries in high-acuity settings.

**Systematic review registration:**

https://www.crd.york.ac.uk/prospero/display_record.php?ID=CRD420251133849, identifier: CRD420251133849.

## 1 Introduction

**Figure 1:**
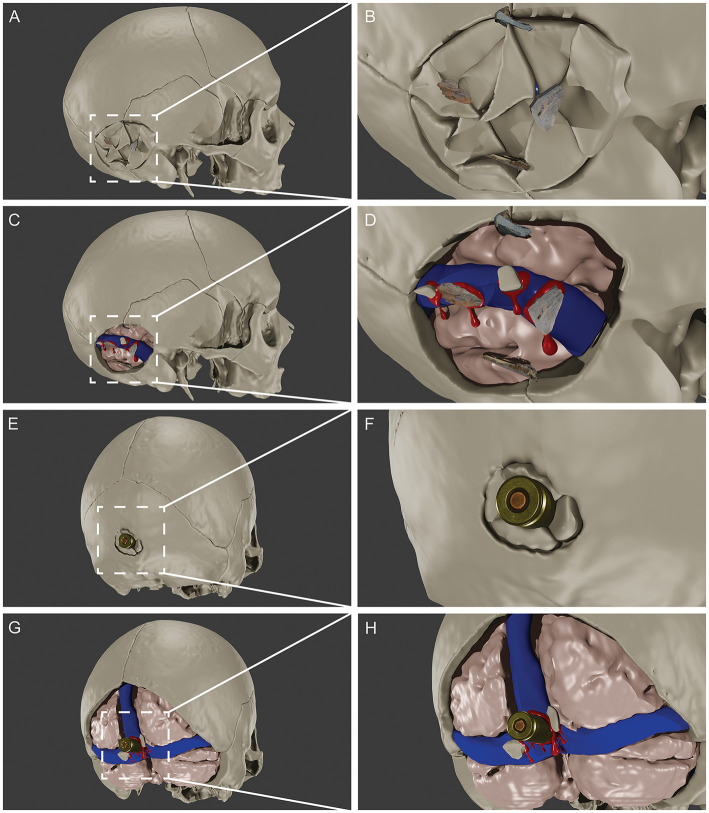
Mechanisms of penetrating dural venous sinus injury. **(A–D)** Missile-generated bone and shrapnel fragments causing sinus laceration. **(A)** Lateral view of a high-energy missile impact producing a comminuted fracture (*dashed box*). **(B)** Close-up of **(A)** showing inward-driven bone fragments. **(C)** Post-craniectomy view showing the injured transverse sinus–sigmoid sinus complex with adherent bone fragments (*dashed box*). **(D)** Close-up of **(C)** showing transverse sinus–sigmoid sinus defect with active hemorrhage. **(E–H)** Penetrating injury from a bullet and associated secondary bone fragments; **(E)** Posterolateral view of occipital bullet entry with a calvarial defect (*dashed box*). **(F)** Close-up of **(E)** showing the entry point of the retained projectile. **(G)** Post-craniectomy view showing the injured torcular Herophili with adherent bone fragments and a projectile (*dashed box*). **(H)** Close-up of **(G)** showing injury of the torcular Herophili with venous disruption and active hemorrhage. SS, sigmoid sinus; TH, torcular Herophili; TS, transverse sinus. Used with permission from Barrow Neurological Institute, Phoenix, Arizona.

Investigation of penetrating injuries to the posterior dural venous sinuses (DVSs), including the sigmoid and transverse sinuses and the torcular Herophili, remains limited (1). These sinuses have a central role in cerebral venous drainage, and given their structural vulnerability, disruption of their integrity has important clinical implications (2–4). The etiological mechanisms leading to DVS injuries are broadly categorized as blunt trauma and penetrating trauma (5). Blunt injuries usually result from forces applied over a broad surface area. In contrast, penetrating injuries disrupt sinus integrity through penetration by foreign objects such as projectiles or secondary missiles, including bone fragments (Figure 1) (6–8). Although penetrating trauma to these venous channels is rare, it is associated with disproportionately high morbidity and mortality. The principal causes of adverse outcomes are uncontrolled hemorrhage, acute venous outflow obstruction, and secondary complications such as venous infarction and cerebral edema (9–11).

Because penetrating posterior DVS injuries are rare and present with heterogeneous initial symptoms, standardized management protocols have been difficult to establish. A systematic synthesis of reported cases is therefore critical to guide neurosurgical decision-making, particularly in high-acuity settings such as trauma centers and military surgical facilities. This review consolidates the available evidence on mechanisms, interventions, and outcomes related to DVS injuries. It also traces the evolution of management strategies from 19th-century rudimentary tamponade techniques to current refined reconstructive approaches.

## 2 Methods

This study was conducted in accordance with the Preferred Reporting Items for Systematic Reviews and Meta-Analyses (PRISMA) guidelines, and it was registered in the PROSPERO international prospective register of systematic reviews (Registration ID: CRD420251133849).

### 2.1 Search strategy

A systematic search was conducted on February 08, 2026, in the PubMed, Scopus, Embase, and Cochrane Library databases, with no time constraints. The following terms were used in advanced searches, and the results were filtered by title and abstract: (((((((((((((((((torcula) OR (torcular Herophili)) OR (confluence of sinuses)) OR (confluens sinuum)) OR (konfluens sinuum)) OR (transverse sinus)) OR (sigmoid sinus)) OR (sinuses, transverse)) OR (transverse sinuses)) OR (lateral sinuses)) OR (lateral sinuses)) OR (sinuses, lateral)) OR (lateral sinus)) OR (sinus, lateral)) OR (sinus transversus)) OR (transversus, sinus)) OR (sinus sigmoideus)) AND (((((penetrant) OR (penetrating)) OR (penetrant injury)) OR (penetrating injury)) OR (injury, penetrating)). Boolean operators “AND” and “OR” were used to combine these Medical Subject Headings (MeSH) terms, creating a comprehensive search strategy. The search query formatting met the advanced search specifications for each database.

### 2.2 Eligibility criteria

For clarity and analytic coherence, we collectively refer to the transverse sinus, sigmoid sinus, and torcular Herophili as the posterior DVSs. Inclusion criteria focused on original articles reporting penetrating injuries to the posterior DVSs. We excluded studies describing blunt trauma or pathological (e.g., tumor), non-penetrating, abutting, or avulsion injuries. Review articles, editorials, commentaries, retracted articles, articles with errata, and non-English studies were also excluded.

### 2.3 Study selection

Search results were initially filtered by title and abstract. Duplicate records were removed. Two independent reviewers (J.T.O., J.E.P.) conducted the screening using the Rayyan platform (12). The selection process followed PRISMA guidelines (13). A supplementary manual search was conducted to identify relevant studies, including a review of the reference lists of all included articles (14). All reviewers (J.T.O., J.E.P., M.G.) reached consensus on all articles included in the study.

### 2.4 Quality assessment and grading of evidence

To assess methodologic quality and potential bias in the included studies, we used the Joanna Briggs Institute Critical appraisal checklist for systematic reviews and research syntheses (15). The overall risk of bias was classified based on the proportion of “yes” items as low (≥70%), moderate (50%−69%), or high (<50%). Two reviewers (K.Q., J.T.O.) independently assessed each study. A third reviewer (J.E.P.) resolved disputes until agreement was reached. We applied the American Association of Neurological Surgeons/Congress of Neurological Surgeons (AANS/CNS) evidence grading system to studies meeting inclusion criteria and classified the evidence as Class I, II, or III (16).

## 3 Results

### 3.1 Study selection

**Figure 2:**
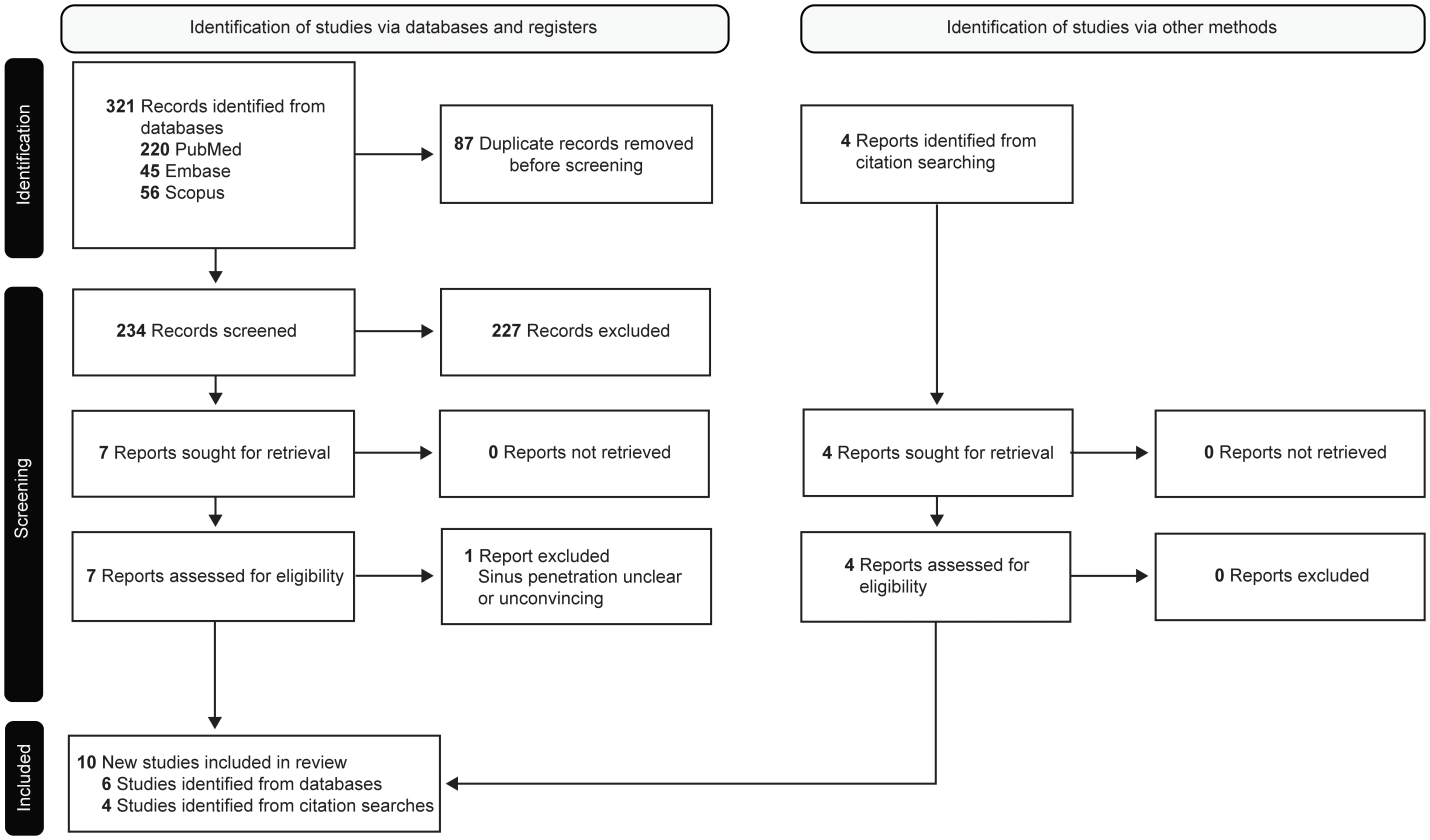
Preferred Reporting Items for Systematic Reviews and Meta-Analyses (PRISMA) flow diagram showing the study selection process. Ten studies that reported penetrating injuries to the posterior dural venous sinuses were identified for inclusion in the review. Used with permission from Barrow Neurological Institute, Phoenix, Arizona.

The systematic search yielded 321 records. After 87 duplicates were removed, the 234 remaining articles were screened by title and abstract. Of these 234 articles, 227 were excluded. The full texts of the remaining seven articles were assessed for eligibility, and one was excluded because it did not definitively confirm sinus penetration. These articles, together with four reports from citation searches, resulted in 10 full-text articles assessed for eligibility. All 10 studies met the inclusion criteria and were included in the review. These included six case reports and four case series, collectively describing 43 patients (Figure 2, Table 1) (9–11, 17–23).

**Table 1:** Summary of penetrating injuries involving posterior DVS.

Sinus	Source	Study design	Setting	No. of posterior DVS injury cases	Patient age, *y*/sex	Mechanism of injury	Initial presentation	Time to surgery	Intervention	Outcome	Key findings
TS	Meirowsky (11)^a^	Case series	Combat	20	NS	MW	GCS NS, varying neurologic deficits (motor, sensory changes), vision impairment common	NS	Sutures, absorbable gelatin sponge (Gelfoam), muscle stamps, ligation	Two deaths; 18 patients survived, but outcomes not further specified	Neurologic deficits cannot be ascribed with certainty to TS injuries
	Kapp and Gielchinsky (17)^a^	Case series	Combat	3	NS	MW	GCS NS	NS	One suture repair, two vein grafts	Suture patent; vein graft one survived	TS injury should be repaired; dominance often unknown
	Morentin and Biritxinaga (23)	Case report	Civilian	1	52/M	MVC	GCS NS, unconscious; sudden hemodynamic deterioration	NA	NA	Death, CTPE	TS laceration may cause CTPE
	Beaty et al. (21)	Case report	Civilian	1	23/M	GSW	Decrease in GCS from 12 to 6, visual deficit, ataxia	Emergent	Suboccipital decompression and sinus ligation	Cognitively intact; persistent visual deficit	Early CTA allows dominance evaluation, enabling safe ligation
	Suhendar et al. (10)	Case report	Civilian	1	8/M	GSW	GCS 14, right zygoma wound	Emergent	Hemostatic agent	GCS 15, no significant neurologic deficits	Hemostatic management preserves venous outflow and yields an intact recovery; prompt repair is key
	Patil et al. (22)	Case report	Civilian	1	13/M	SW	GCS 15, no deficits	Emergent	Dural hitch sutures	Uneventful recovery	Nonsupine intubation; needed to avoid sinus shear; maintain CPP
SS	Meirowsky (11)^a^	Case series	Combat	4	NS	1 GSW, 3 MW	GCS NS, peripheral facial palsy, sensorineural hearing loss, cerebellar deficits	NS	One suture, two muscle stamps, one silver clip + Gelfoam	No fatalities, significant and permanent cerebellar deficits, facial palsy, and deafness	High rate of morbidity despite survival; infections not observed
	Paluszkiewicz et al. (19)	Case report	Civilian	1	47/M	MVC	Decrease in GCS from 13 to 5, ↑ICP, subtentorial herniation	Emergent	Direct packing, graft, rfVIIa,	Brain death	Direct packing + rfVIIa is a promising method to control sinus hemorrhage
TH	Meirowsky (11)^a^	Case series	Combat	1	NS	MW	GCS NS	NS	NS	Recovered; no motor or visual deficit	Although extremely rare, isolated TH injuries can be survivable
	Pricola et al. (20)	Case report	Civilian	1	21/M	GSW	GCS 10, hemorrhage	Emergent	Suture + graft	Full recovery; no deficits; discharged POD 3	Timely torcular repair preserves patency and neurologic function
TS + SS	Cushing (18)	Case series	Combat	1	NS	MW	GCS NS, visual loss, dullness, brisk reflexes, nystagmus	24 h	TS clip; conservative management for SS injury	Survived; left hemianopsia, right-sided deafness	Conservative retention of a tamponading SS bone fragment averted fatal hemorrhage
	Meirowsky (11)^a^	Case series	Combat	1	NS	MW	GCS NS	NS	NS	Death after pulmonary complications	Systemic complications may arise despite initial hemorrhage control
	Abdel-Aziz and Ragaee (9)	Case series	Civilian	2	37/M, 5/F	Fracture	M: GCS 12, hemorrhagic contusions F: GCS 8, suboccipital hemorrhage	Emergent	Hemorrhage control; ligation attempted	Both patients died of hypovolemic shock	High risk of hemorrhage favors a conservative approach over surgical intervention
TS + TH	Meirowsky (11)^a^	Case series	Combat	5	NS	MW	GCS NS, varying levels of consciousness; predominant deficit was visual impairment that ranged from light perception to initial blindness	NS	Sutures; Gelfoam, muscle stamps	No deaths; visual recovery in most; one retained hemianopia	Temporary TH occlusion is tolerable; reestablishing venous flow is recommended via repair after thrombus removal

CPP, cerebral perfusion pressure; CTA, computed tomography angiography; CTPE, cerebral tissue pulmonary embolization; DVS, dural venous sinus (or sinuses); GCS, Glasgow Coma Scale score; GSW, gunshot wound; ICP, intracranial pressure; MVC, motor vehicle crash; MW, missile wound; NA, not applicable; NS, not specified; POD, post-operative day; rfVIIa, recombinant factor VIIa; SS, sigmoid sinus; SW, stab wound; TH, torcular Herophili; TS, transverse sinus; ↓, decreased; ↑, elevated; +, plus.

a Some historical sources lacked sufficient detail to disaggregate data at the individual patient level.

### 3.2 Quality assessment and grading of evidence

Seven studies (70%) were at low risk of bias, three (30%) at moderate risk, and none at high risk. Common limitations included incomplete or unclear reporting, heterogeneous definitions of interventions, and limited outcome data (Supplementary Table 1) (9–11, 17–23). All 10 included studies (100%) provided Class III evidence (Supplementary Table 2) (16).

### 3.3 Patient demographics and context

**Figure 3:**
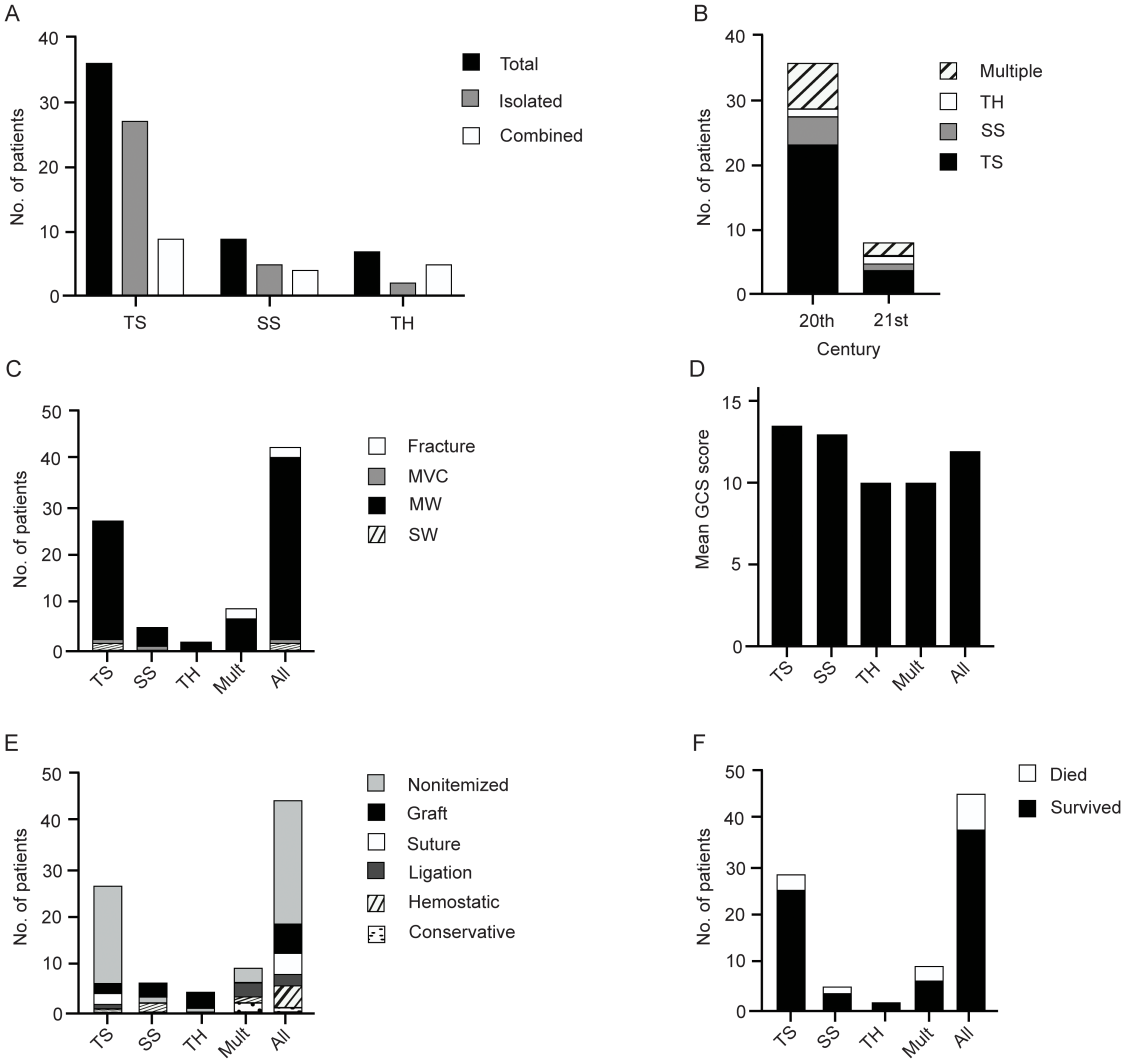
Quantitative analysis of penetrating posterior dural venous sinus injuries from the 43 reported cases. **(A)** Frequency of injuries to an isolated sinus vs. injuries to multiple sinuses by sinus. **(B)** Distribution of reported cases in the 20th and 21st centuries. Stacked bars display case counts by injured sinus. **(C)** Frequency of mechanism of injury by sinus. Stacked bars show relative frequency of mechanism of injury. **(D)** Mean GCS score by location of injury. **(E)** Distribution of interventions by injured sinus. Stacked bars show relative frequency of intervention. **(F)** Mortality rate by sinus. These data are also summarized in Supplementary Table 3. All, entire cohort; GCS, Glasgow Coma Scale; Mult, multiple (i.e., >1 sinus involved); MVC, motor vehicle crash; MW, missile wound; SS, sigmoid sinus; SW, stab wound; TH, torcular Herophili; TS, transverse sinus. Used with permission from Barrow Neurological Institute, Phoenix, Arizona.

Age and sex were reported for eight of 43 patients (18.6%). In this subset, the mean (SD) age was 25.8 (16.6) years (range, 5–52 years); seven patients (87.5%) were male, and one (12.5%) was female. Of 43 cases, 34 (79.1%) were isolated injuries to a single sinus, and nine (20.9%) involved combined injuries to multiple sinuses (Figure 3A). Of the 43 patients, 35 (81.4%) were reported in 20th-century wartime sources, and eight (18.6%) in 21st-century civilian reports (Figure 3B).

### 3.4 Mechanism of injury

The included articles described four primary mechanisms of penetrating injury: missile wounds, fractures, motor vehicle crashes, and stab wounds. Missile wounds were the predominant mechanism of injury across all sinus types, accounting for 38 of the 43 cases (88.4%) in which a mechanism was specified (Figure 3C). Specifically, 25 of 27 isolated transverse sinus injuries (92.6%), four of five isolated sigmoid sinus injuries (80%), two of two isolated torcular Herophili injuries (100%), and seven of nine (77.8%) injuries to multiple sinuses were attributed to missile wounds. Only two injuries (4.7%) were due to motor vehicle crashes, one to the transverse sinus and one to the sigmoid sinus. A single stab wound accounted for one transverse sinus injury (2.3%). Fractures were the cause in two cases (4.7%), both of which involved multiple sinuses.

### 3.5 Clinical presentation and outcomes

#### 3.5.1 Isolated transverse sinus injuries

The most frequently injured sinus, the transverse sinus, was involved in 36 of the 43 cases (83.7%) (27 isolated, nine multiple sinuses). Of the isolated injuries, 23 were combat-related (85.2%) and four were civilian (14.8%). Missile wounds accounted for most isolated transverse sinus injuries (25 of 27, 92.6%), followed by a single case each of an motor vehicle crash (3.7%) and a stab wound (3.7%).

Clinical presentations were reported heterogeneously. Glasgow Coma Scale (GCS) scores were reported for three of 27 (11.1%) and were not specified for 24 patients (88.9%; Figure 3D). Among the three patients with reported GCS scores, initial scores ranged from 12 to 15, with a median of 13. One patient developed visual deficits and ataxia; this patient also had a concurrent decrease in GCS score from 12 to 6. Of the 24 patients without GCS values, one described unconsciousness with sudden hemodynamic collapse.

Interventions were described in 26 of 27 transverse sinus injuries (Figure 3E). Reported management strategies for isolated cases included suture repair (*n* = 1; survival with a patent lumen), vein grafting (*n* = 2; one survival, one outcome not specified), hemostatic agent (*n* = 1; survival), dural hitch suture (*n* = 1; survival), and sinus ligation (*n* = 1; survival with persistent visual deficit). The remaining 20 cases came from a wartime series that described sutures, an absorbable gelatin sponge (Gelfoam), muscle stamps, and ligation but lacked per-patient attribution. The mortality rate was three of 27 (11.1%): two deaths occurred in the 20-case wartime series (not attributable to technique), and one death occurred due to post-traumatic cerebral venous thromboembolism in the case report that did not specify an intervention (Figure 3F).

#### 3.5.2 Isolated sigmoid sinus injuries

Nine cases (20.9%) involved the sigmoid sinus, and five of the nine were isolated and four involved injuries to multiple sinuses. Isolated sigmoid sinus injuries occurred in four combat cases and one civilian case. Mechanisms of injury were reported for all five isolated sigmoid sinus cases: missile injuries in four (80.0%) and trauma from a motor vehicle crash in one (20.0%; Figure 3C). However, clinical presentations were heterogeneous. A GCS score was documented in only one of the five isolated sigmoid sinus cases (20.0%) and was not specified in the remaining four (Figure 3D). In the case in which a GCS score was reported, the score decreased from 13 to 5, with increased intracranial pressure and subtentorial herniation. For the remaining four cases, narrative descriptions included peripheral facial palsy, sensorineural hearing loss, and cerebellar deficits.

Interventions were described in all cases. Reported management strategies included packing with a graft and hemostatic agent (*n* = 1; death), suture repair (*n* = 1; survival), muscle stamp (*n* = 2; survivals), and silver clip with Gelfoam (*n* = 1; survival). Overall mortality was one in five cases (20.0%; Figure 3F).

#### 3.5.3 Isolated torcular Herophili injuries

Seven cases (16.3%) involved the torcular Herophili; of these, two were isolated and five occured in multiple sinuses. Of the isolated cases, one was wartime and one was civilian. Mechanisms were reported for both isolated torcular Herophili cases, and both were missile injuries. A GCS score was explicitly reported in one case (score, 10; survival) and not reported in the other; in the case that did not report GCS score, narrative descriptions noted stable vital signs with wound hemorrhage. Interventions were performed in one case (suture with synthetic graft) but were unspecified in the other. Both patients survived (Figure 3F).

#### 3.5.4 Injuries involving multiple sinuses

Combined transverse and sigmoid sinus injuries were identified in four of 43 cases (9.3%): two military and two civilian patients. Mechanisms were reported in all four cases, with two missile wounds and two fracture-related injuries. GCS scores were reported in two cases (12 and 8) and not reported in the remaining two (Figure 3D). Interventions were reported in three of the four cases (Figure 3E). These included hemorrhage control with ligation attempts (*n* = 2) and transverse sinus clipping with conservative management of the sigmoid sinus injury (*n* = 1). Overall mortality was high (3 of 4, 75.0%), consisting of two deaths from hypovolemic shock after ligation attempts and one death from pulmonary complications in the case with an unspecified intervention (Figure 3F).

Injuries to both the transverse sinus and torcular Herophili were reported in five of 43 cases (11.6%), all occurring in wartime settings from missile wounds. Although GCS scores were not documented, narrative descriptions indicated variable levels of consciousness, with visual impairment ranging from light perception to complete blindness. Interventions were described in every case and included sutures, Gelfoam, and muscle stamps. Notably, no mortality was recorded (Figures 3E, F).

## 4 Discussion

Traumatic injuries to the DVSs are uncommon yet potentially devastating in both civilian and military neurotrauma, with reported proportions ranging from 1 to 4% in civilian settings and 4 to 12% in wartime (1). The primary dangers of these injuries include: (1) life-threatening hemorrhage from low-pressure, high-volume venous channels; (2) venous air embolism; and (3) venous outflow obstruction, which can trigger a cascade of secondary insults, including DVS thrombosis, elevated intracranial pressure, cerebral edema, and hemorrhagic venous infarction (24–26).

The etiology of DVS injury is diverse. Blunt trauma is the most common cause in civilian settings; it typically involves depressed or linear skull fractures that compress the sinus or create an endothelial nidus for thrombosis in an underlying sinus (27–29). Conversely, penetrating injuries result from direct violation of a sinus by a foreign body, such as a projectile or secondary sharp bone fragments (25, 30). This mechanism is associated with high morbidity and mortality because of direct tissue destruction, contamination, and complex hemorrhage control (26). This study synthesizes the literature on penetrating posterior DVS injuries, a cohort largely shaped by historical military reports. Hence, a review of the historical context is warranted to elucidate the evolution of management strategies before undertaking a sinus-specific analysis.

### 4.1 Historical context of DVS injury and repair

The surgical management of DVS injuries has evolved over nearly two centuries, with early insights often drawn from isolated case reports. As early as 1828, Benjamin Brodie noted that the transverse and superior sagittal sinuses were frequently affected by trauma (31, 32). By the mid-19th century, surgeons began documenting more direct management of hemorrhage from these sinuses. In the latter part of the 19th century, advances in surgical techniques were made, and an understanding of asepsis emerged (33). In 1888, Charles Nancrede advocated conservative measures such as sponge packing and antiseptic tampons, suggesting they could provide temporary and often permanent control of hemorrhage (34).

The management of penetrating sinus injuries has advanced through military conflicts. During World War I, Harvey Cushing, Percy Sargent, and Gordon Holmes described the clinical course and surgical treatment of these injuries (18, 35, 36). Cushing was cited as stating that early surgical intervention might prevent progressive venous thrombosis, a critical insight given his own review of cases showing a 79% mortality rate for DVS injuries at that time (36).

**Figure 4:**
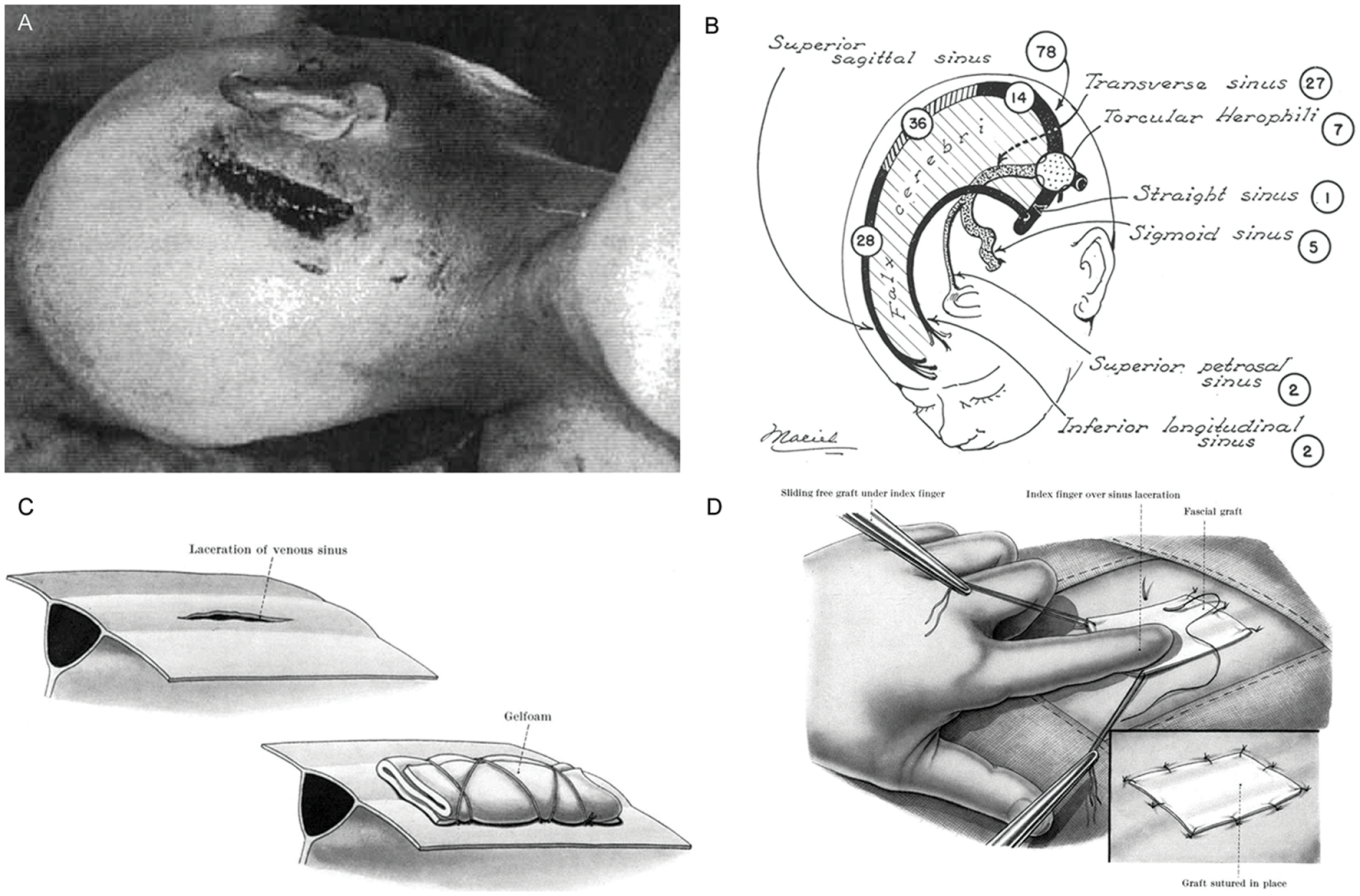
Historical depictions of dural venous sinus injuries. **(A)** Clinical photograph from a report by Cushing (18) from the World War I era showing a shell fragment wound that created a gutter fracture, which injured the transverse and sigmoid sinuses. **(B)** Distribution of patients with dural venous sinus (DVS) wounds during the Korean War, as described by Meirowsky (11). The schematic shows the number of operative cases by sinus treated by U.S. Army units. **(C)** Overlay repair of a DVS laceration using absorbable gelatin sponge (Gelfoam) and figure-eight sutures, as described by Kempe (38). At top is a linear laceration of a DVS; at bottom, the laceration is repaired with a free muscle graft or an absorbable gelatin sponge (Gelfoam) laid over the defect and secured with interrupted figure-eight fine silk sutures placed lateral to the sinus margins. **(D)** Underlay graft reconstruction of a DVS laceration, also from the description by Kempe (38). With the assistant's index finger covering the laceration, a pericranial or temporalis fascial graft is slid beneath and is sutured in place (inset) to cover the defect and restore its patency (38). **(A)** is used with permission from Cushing (18). **(B)** is used with permission from Meirowsky (11). **(C, D)** are used with permission from Kempe (38).

After World War II, Donald Matson outlined surgical recommendations for the closure of DVS defects and for hemostasis (37). Experience from the Korean War, as reported by Meirowsky (11), led to a detailed classification of DVS wounds, although Meirowsky concluded that techniques for functional restoration of vascular continuity were limited at the time (Figure 4) (11, 17, 18, 38). Subsequent experience during the Vietnam War led to more aggressive attempts at DVS reconstruction aimed at preserving blood flow, as reported by Kempe (38). William Hammon advocated a method to create space by placing burr holes around the periphery of the penetration and performing a circumferential craniectomy to prevent catastrophic hemorrhage from a DVS during exposure (39). John Kapp and Isaac Gielchinsky emphasized hemorrhage control, using internal shunts and Fogarty balloon catheters for temporary endoluminal occlusion (17, 40). Surprisingly, no reports were identified from more recent conflicts, although this may reflect a lack of acceptable or detailed information, increased rates of immediate death due to increased weapon lethality, and the specific anatomical region involved.

### 4.2 Injuries of the dural venous sinuses: mechanisms, clinical features, and surgical management

#### 4.2.1 Transverse sinus

The transverse sinus is the primary conduit for venous outflow from the torcular Herophili and the second-most common site of traumatic DVS injury after the superior sagittal sinus (26, 28). In our study, the transverse sinus was the most frequent site of posterior penetrating DVS injury (84%). The clinical presentation of transverse sinus injury is primarily determined by the mechanism of injury. The penetrating injuries identified in our study, primarily missile wounds, resulted in direct lacerations presenting as an acute hemorrhagic crisis. In contrast, blunt trauma from occipital bone fractures typically initiates a more insidious pathological cascade of endothelial injury that leads to progressive thrombosis (27, 28). This thrombosis can produce a delayed syndrome of venous hypertension with non-specific symptoms of headache, papilledema, and visual disturbances, which were first documented well with direct sinography (28). Diagnosis has evolved from invasive historical techniques to modern non-invasive modalities, including computed tomography (CT), venography, and magnetic resonance venography. These imaging modalities not only confirm thrombosis or laceration but also crucially determine sinus dominance and inform surgical intervention (24, 26).

The management of transverse sinus injuries is a high-stakes decision between preserving or sacrificing the sinus. Among the cases included in our review, one patient with transverse sinus ligation survived but had a persistent visual deficit. This case highlights the risks inherent in the sacrifice of a functionally critical sinus. For repairable lacerations, modern options include direct suture, vein grafts, or a reflected dural flap, with the latter particularly effective for the treatment of complex iatrogenic injuries at the transverse sinus-sigmoid sinus junction (41).

For post-traumatic thrombosis without laceration, a different therapeutic paradigm applies. Systemic anticoagulation with heparin is often used to prevent clot propagation and can result in complete recanalization (42, 43). In severe cases of thrombosis with refractory elevation of intracranial pressure, endovascular thrombolysis with agents such as urokinase has been successfully used as a salvage therapy, resulting in sinus recanalization and rapid neurologic improvement (44).

The prognosis for transverse sinus injury remains guarded because of the critical role of this sinus in venous drainage. In this review, isolated penetrating transverse sinus injuries had an 11% mortality rate, considerably lower than the 41.7% mortality rate reported in a large mixed-mechanism trauma database. This discrepancy may reflect differences in patient populations and the focused, damage-control context of military surgical practice (45).

#### 4.2.2 Sigmoid sinus

Due to its complex, curved course through the petromastoid temporal bone, the sigmoid sinus is vulnerable to a spectrum of injuries. Its proximity to the mastoid air cells provides a pathway through which fractures can introduce air directly into the sinus. The presence of intrasinus gas is not an incidental finding but a strong predictor of subsequent DVS thrombosis, with an odds ratio of 11.3. This is likely because the same endothelial injury that allows air to enter also serves as a potent nidus for clot formation (46).

The clinical presentation of a sigmoid sinus injury is highly context-dependent (30). In blunt trauma, sigmoid sinus injury may present insidiously, as in one case in our review in which a patient with a closed head injury developed delayed hearing loss and tinnitus, ultimately attributed to a thrombosed sigmoid sinus (42). Our study results also highlight that penetrating injuries to the sigmoid sinus often present with acute cranial nerve deficits. The trajectory of a penetrating object that injures the sigmoid sinus almost invariably places the facial and vestibulocochlear nerves at risk within the temporal bone, explaining the high rates of facial palsy and deafness (25).

Management principles for sigmoid sinus injury are best illustrated by contrasting iatrogenic and penetrating trauma. The meticulous, size-based repair algorithm for iatrogenic injuries uses bipolar coagulation for pinholes, direct microsutures for lacerations less than 3 mm, and a sutureless only patch for larger defects that cannot be sutured; these techniques yield excellent outcomes with long-term patency (30). The marked disparity between the favorable outcomes of iatrogenic injury series and the 20% mortality rate observed in penetrating injuries underscores that injury context and energy are as critical as the anatomical injury itself. Although iatrogenic injuries are generally clean and controlled, penetrating injuries typically produce high-energy, contaminated wounds that mandate damage-control management (30).

Survivors of penetrating sigmoid sinus injuries in this study often had substantial cranial nerve morbidity. By contrast, blunt trauma–induced thrombosis carries a more favorable prognosis when managed with timely anticoagulation or complete sinus recanalization, and symptoms can fully resolve (42).

#### 4.2.3 Torcular Herophili

Injury to the torcular Herophili is rare (25). In this study, two cases were identified, both resulting from high-velocity missile wounds that directly penetrated the sinus. Obstruction at this critical junction compromises nearly all cerebral venous drainage and precipitates catastrophic intracranial hypotension.

An unexpected insight from this review is the relative resilience of the torcular Herophili despite its central role in cerebral drainage. Along with recent case reports, this review highlights a critical dichotomy in the presentation of different types of injuries to the torcular Herophili (11, 20). As seen in some cases in this study, direct laceration from a projectile may cause an acute hemorrhagic crisis. Alternatively, extrinsic compression from an inwardly displaced bone fragment due to a nearby gunshot wound may present as a subacute syndrome of progressive intracranial hypertension with worsening diplopia (27). CT and CT venography are therefore essential to distinguish these two entities.

The surgical management of a torcular Herophili injury must be precisely tailored to the specific insult. Management of any torcular Herophili injury must be aggressive and aimed at preserving or restoring flow; ligation is strongly discouraged (26). In the reviewed cases, direct lacerations were successfully repaired with suture and synthetic graft reconstruction, resulting in full recovery. For compressive injuries, the surgical goal is not sinus wall repair but rather sinus decompression. The surgical technique is to perform a circumferential so-called “doughnut craniectomy” to isolate the depressed fragment and then carefully dissect it away from the intact dura (27).

Despite the critical role of the torcular Herophili, the successful outcomes reported for both direct repair and decompression convey an important message. With timely diagnosis and meticulous, mechanism-appropriate surgical technique, neurologic function can be preserved even after injury to one of the most critical points of the DVS system.

### 4.3 Tailoring surgical strategy to extent of injury

In penetrating injuries, in addition to the mechanism of injury, the extent of the penetrating posterior DVS injury is also critical for choosing the suitable repair technique (Table 2) (9–11, 17–22). For consistency, injuries are classified here as focal rents (≤5 mm), focal wall defects (5–10 mm), patchable wall loss, segmental and destructive wall loss (about >1 cm or shredded margins), complete transection, and junctional injuries.

**Table 2:** Successful vs unsuccessful management strategies of penetrating posterior DVS injuries, stratified by injury extent and mechanism.

Sinus	Mechanism of injury	No. of patients	Injury extent	Successful management	Unsuccessful management
Transverse	SW (22)	1	Focal linear defect	Dural hitch sutures	No
	GSW (10)	1	Penetrating track, limited wall loss	Hemostatic wrap and closure	No
	MW (11)	27	Patchable laceration	Primary repair (pledgeted sutures); muscle stamp	NS
	MW (17)	2	Segmental wall loss, destructive defect	Interposition vein graft	Interposition vein grafts (thrombosis reported in transverse sinus)^a^
	GSW (21)	1	Complete transection	Ligation	No
Sigmoid	MVC (19)	1	Focal wall defect	No	Direct packing, graft, rfVIIa
	MW (11)	5	Patchable laceration	Primary repair (interrupted sutures); clip + absorbable gelatin sponge (Gelfoam); muscle stamp	No
Torcular Herophili	GSW (20)	1	Composite focal defect	Hybrid repair: inferior edge primary repair with sutures; superior edge patch graft	No
Transverse + sigmoid	MW (18)	1	Junctional tear	Retention of tamponading bone fragments in sigmoid sinus and transverse sinus clip	No
	Fracture (9)	2	Combined junctional disruption	No	Hemorrhage control; rostral ligation attempted
Transverse + torcular Herophili	MW (11)	5	Junctional tear	Primary repair (pledgeted sutures); muscle stamp	NS

DVS, dural venous sinus; GSW, gunshot wound; MW, missile wound; MVC, motor vehicle crash; NS, not specified; rfVIIa, recombinant factor VIIa; SW, stab wound; +, plus.

a Two interposition saphenous vein grafts were reported: one graft was patent with patient survival, and the other thrombosed and led to death.

#### 4.3.1 Transverse sinus

For penetrating transverse sinus injuries, a stepwise approach based on injury extent is helpful; it progresses from focal repair to reconstruction as tissue loss increases. Focal rents can be controlled with dural hitch (tack-up) sutures that anchor the dura to the inner table, compressing the defect without stenosis (22). Penetrating tracks with a retained missile can be managed with temporary proximal and distal transverse sinus control; an approximately 1 cm longitudinal sinusotomy to expose and extract the foreign body, followed by hemostatic agents; and then unclamping once hemostasis is confirmed (10).

In patchable wall losses, linear or stellate lacerations with edges that can be approximated can be effectively repaired with interrupted non-absorbable venorrhaphy, with bites tied over Gelfoam and fibrin pledgets to seal the line (11, 17). Larger tears can be repaired with an autologous muscle stamp sized about twice the laceration width, a small intraluminal tuck, a few peripheral silk stitches, and hemostatic pledgets (11).

In transection and segmental loss, when sacrifice is acceptable (e.g., codominant transverse sinus), surgeons may perform unilateral ligation after wide suboccipital exposure (21). When preservation is required, interposition saphenous vein grafting is performed with an internal shunt and Fogarty control. The procedure begins with wide exposure, followed by clot evacuation and irrigation with heparinized saline. Frayed intimal edges are then carefully debrided. Anastomosis is initiated with non-absorbable continuous sutures along one wall, and the opposite wall is partially closed. Once the shunt is removed, the anastomosis is completed by tying down the pre-placed interrupted sutures (17). However, failures with transverse sinus vein grafts (graft thrombosis) have been reported, and fatal courses with unspecified management are described for blunt transverse sinus tears (17, 23).

#### 4.3.2 Sigmoid sinus

For a focal wall breach with high-flow hemorrhage, packing is made effective through mechanical fixation. A fibrin sponge is pressed into the defect and secured with a gauze strap; the dural roof is covered with a temporalis muscle onlay, which is fixed with a strap-fixation device (19). In particular, strap-fixed packing, combined with recombinant factor VIIa, has been associated with poor overall outcomes in one case reported in the literature (19). For lacerations amenable to patch repair, management may involve direct interrupted suturing (when margins permit), muscle stamps for larger defects, or silver clips, typically supported by a Gelfoam buttress applied to the injured wall. However, loose, unsupported packing should be avoided in favor of strap-fixed tamponade or definitive repair (11).

#### 4.3.3 Torcular Herophili

Reports of penetrating injuries to the torcular Herophili are sparse and limited, as they do not provide sufficient variation in injury extent to allow categorization as focal, segmental, or complete transections. A case report describes a composite focal defect managed in a component-wise fashion (20). The inferior 5-mm segment can be repaired primarily with a running nylon suture, whereas the superior 10-mm segment, not amenable to primary closure, can be reconstructed using a synthetic dural graft secured with sutures. During peak hemorrhage, elevating the head of the bed to about 60° for several minutes can reduce venous return. Continuous-wave Doppler ultrasonography is also useful for the early detection of venous air emboli in the torcula and for confirming that venous outflow is restored after reconstruction (20).

Historical reports of posterior DVS repairs at this junction also describe silk sutures tied over Gelfoam pledgets and muscle stamps for abraded, multipoint oozing surfaces (11). Although current literature emphasizes the importance of reconstruction in penetrating torcular injuries, reports of failed repairs remain limited (11, 20).

#### 4.3.4 Injuries to multiple sinuses

At the transverse-sigmoid sinus junction, surgeons may retain any existing tamponade (e.g., a tamponading bone fragment *in situ*) and apply a local silver clip to the torn wall (18). Additional hitching stitches from adjacent dura to the skull provide compression without narrowing outflow (18). In exsanguinating scenarios, rostral ligation has been attempted but is associated with poor outcomes in contemporary series (9). Linear tears at the transverse sinus–torcular Herophili junction with salvageable edges can be managed with primary repair (interrupted sutures tied over Gelfoam and fibrin). Larger junctional rents are reinforced with a muscle stamp, contoured to preserve patency at the torcular Herophili (11).

### 4.4 Repair principles and post-operative management of penetrating DVS injury

Overall, the modern management of penetrating DVS injuries has evolved from a reactive focus on hemorrhage control to a strategy centered on preserving venous function. The critical first step is advanced vascular imaging. Either CT venography or magnetic resonance venography is essential to precisely define the injury and delineate the patient's unique venous anatomy and sinus dominance (24, 27). This knowledge shifts the surgical approach from reactive improvisation to an anatomically informed plan; it prioritizes wide exposure and hemorrhage control to secure proximal and distal sinus segments before the injury is addressed (25, 26).

The repair follows a graded, reconstructive approach. The choice of technique is tiered based on the defect's complexity (25, 30, 41). Minor defects may be managed with simple tamponade or bipolar coagulation; clean, linear tears are ideally suited for direct microsuturing. Complex defects require patch reconstruction with autologous or synthetic materials. Segmental destruction of a critical sinus necessitates interpositional grafting, most commonly with a reversed saphenous vein segment.

**Figure 5:**
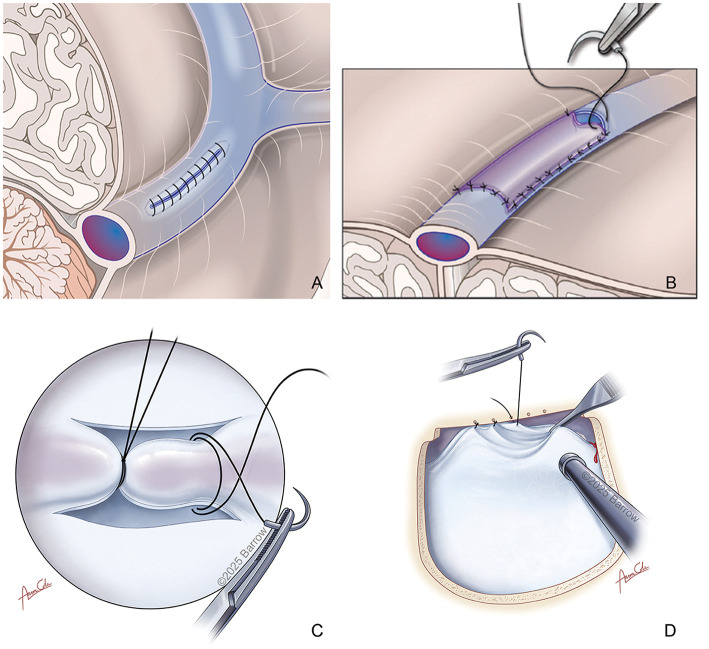
Illustrations of primary surgical repair techniques for penetrating dural venous sinus (DVS) injuries, as described by Davidson and Armonda (48). **(A)** Direct primary suture repair of a linear laceration (48). **(B)** Repair of a larger defect using a patch graft fashioned from pericranium or fascia, which is sutured into place to restore luminal integrity (48). **(C)** Ligation of the transverse sinus. After mobilization of the sinus, two circumferential sutures are placed proximal and distal to the laceration to achieve hemostasis. **(D)** Dural tenting and a tack-up suture anchored through the overlying bone to elevate and compress a laceration in the sinus. **(A, B)** are used with permission from Richards and Harrigan (49). **(C, D)** are used with permission from Barrow Neurological Institute, Phoenix, Arizona.

**Figure 6:**
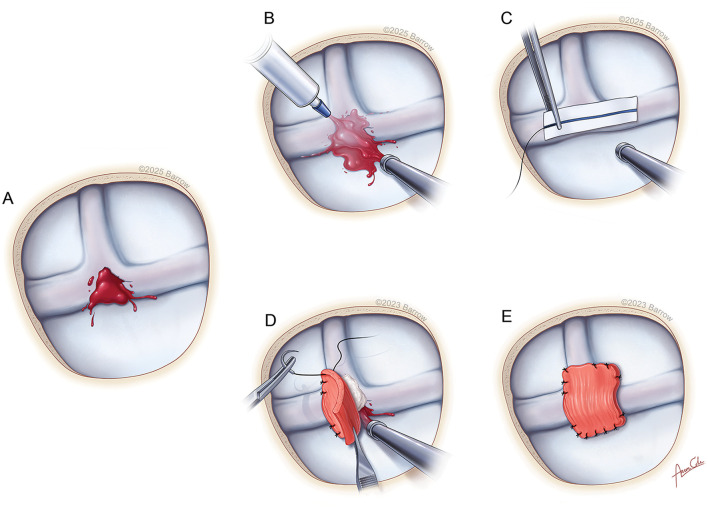
Repair techniques for penetrating torcular Herophili (TH) injuries. **(A)** Injury to the torcular Herophili. **(B)** Irrigation of the wound, followed by **(C)** direct tamponade with a hemostatic agent. **(D)** Repair via a patch of autologous muscle. **(E)** The completed repair. Used with permission from Barrow Neurological Institute, Phoenix, Arizona.

Finally, vigilant post-operative management is crucial. Delayed thrombosis at the repair site remains a major concern (28, 44). The role of post-operative anticoagulation is highly controversial; although it is beneficial in treating established DVS thrombosis, it must be used with extreme caution in acute penetrating trauma with associated intracranial hemorrhage (43, 44, 47). A tiered reconstructive philosophy tailored to the specific injury, followed by careful post-operative monitoring, offers the best chance for preservation of neurologic function after these challenging injuries (Figures 5, 6) (48).

### 4.5 Future directions and limitations

Despite being recognized for almost 200 years and managed with various surgical techniques, penetrating DVS injuries continue to carry high morbidity and mortality. Future research should focus on prospective data collection, advanced imaging for pre-operative assessment, strategies to reduce the risk of venous sinus thrombosis, and the role of systemic anticoagulation or antiplatelet therapy after surgical repair.

Limitations of this review include reliance on heterogeneous case reports and series, which precluded patient-level analyses and confined outcomes to aggregate data. Additionally, the absence of controlled comparisons and the heterogeneity of surgical approaches further limited the generalizability of the conclusions. Inconsistent reporting of anatomical dominance limited insights regarding flow sparing, whereas survivorship bias likely underestimated the true lethality of these injuries. Publication bias is also probable because of a tendency to report unusual or successful cases and to underreport negative or non-operative outcomes. Together, these limitations could lead to an overestimation of the benefit of flow-preserving strategies and an underestimation of complications. Finally, most published cases involved combat settings, which may limit generalizability to civilian injuries.

## 5 Conclusion

This review of 43 patients with penetrating posterior DVS injuries highlights several key insights. For such severe injuries requiring complex surgical and critical care, detailed case information is sparse, likely related to the initial devastating insults resulting in high rates of mortality or extreme morbidity and the rapidity in which treatment must be instituted. Transverse sinus injuries occur more frequently than torcular Herophili and sigmoid sinus injuries, and outcomes are heterogeneous, influenced by dominance and timely hemostasis. Transverse sinus repair was commonly performed with sutures and grafts. Conversely, sigmoid sinus injuries often required damage control with tamponade. However, for torcular Herophili injuries, the evidence supports reconstructive techniques to preserve venous outflow, which was associated with improved survival in the reported cases.

Three principles guide the successful management of penetrating posterior DVS injuries. First, determine venous dominance pre-operatively or intraoperatively, and prioritize preservation of the dominant side, especially at the transverse sinus. Reserve ligation of the transverse sinus for clearly codominant or contralaterally dominant systems. Second, when feasible, prioritize reconstructive techniques that preserve venous patency, especially at the torcular Herophili and at the dominant or codominant transverse sinus. Third, for sigmoid sinus injuries with hostile exposure and difficult reconstruction, consider hemostasis with tamponade or selective ligation.

## Data Availability

The original contributions presented in the study are included in the article/Supplementary material, and further inquiries can be directed to the corresponding author.
