# Supplementary material for: Penetrating injuries of the posterior dural venous sinuses: a systematic review of injury mechanisms and repair techniques
Source: Front Neurol. 2026 Jul 29;17:1827710. doi: 10.3389/fneur.2026.1827710 (PMC13461576; doi:10.3389/fneur.2026.1827710)
Supplement: Supplementary file 1 [file Supplementary_file_1.docx]

**SUPPLEMENTAL MATERIALS**

**Supplemental Table 1.** JBI risk of bias quality assessment^a^

| **Study** | **Q1** | **Q2** | **Q3** | **Q4** | **Q5** | **Q6** | **Q7** | **Q8** | **Q9** | **Q10** | **Yes rate, %** | **Risk** |
| --- | --- | --- | --- | --- | --- | --- | --- | --- | --- | --- | --- | --- |
|  |  |  |  |  |  |  |  |  |  |  |  |  |
| Cushing, 1917 (1) | Yes | Yes | Yes | Yes | Yes | Yes | Yes | No | – | – | 88 | Low |
| Meirowsky,1959 (2) | Yes | Yes | U | Yes | Yes | No | U | U | Y | NA | 50 | Mod |
| Kapp and Gielchinsky, 1972 (3) | Yes | Yes | U | Yes | Yes | No | No | Yes | Yes | NA | 60 | Mod |
| Morentin and Biritxinaga, 2006 (4) | Yes | Yes | Yes | Yes | NA | NA | NA | Yes | – | – | 63 | Mod |
| Paluszkiewicz al., 2008 (5) | Yes | Yes | Yes | Yes | Yes | Yes | Yes | Yes | – | – | 100 | Low |
| Pricola et al., 2011 (6) | Yes | Yes | Yes | Yes | Yes | Yes | Yes | Yes | – | – | 100 | Low |
| Beaty et al., 2012 (7) | Yes | Yes | Yes | Yes | Yes | Yes | Yes | Yes | – | – | 100 | Low |
| Abdel-Aziz and Ragaee, 2019 (8) | Yes | Yes | Yes | Yes | Yes | Yes | Yes | Yes | N | NA | 80 | Low |
| Suhendar et al., 2022 (9) | Yes | No | Yes | Yes | Yes | Yes | No | Yes | – | – | 75 | Low |
| Patil et al., 2024 (10) | Yes | Yes | Yes | Yes | Yes | No | Yes | Yes | – | – | 88 | Low |
|  |  |  |  |  |  |  |  |  |  |  |  |  |

Abbreviations: JBI, Joanna Briggs Institute; Mod, moderate; NA, not applicable; U, unclear; –, question not available for this article type.

^a^Q1–Q10 refer to questions 1 through 10 from the JBI critical appraisal checklist, which applies to either case reports or case series depending on the specific study. The risk of bias was rated as low if 70% or more of the checklist items were met, moderate if 50% to 69% were met, and high if fewer than 50% were met. Grading was derived from Zachary M, Barker TH, Moola S, et al. Methodological quality of case series studies: an introduction to the JBI critical appraisal tool. *JBI Evid Synth.* 2020;8(10).

**Supplemental Table 2.** American Association of Neurological Surgeons/Congress of Neurological Surgeons (AANS/CNS) Classification of Evidence

| **Class** | **Evidence** | **Recommendations** |
| --- | --- | --- |
| I | Rigorously designed randomized controlled trials or systematic reviews and meta-analyses of such trials | Interventions supported can be strongly recommended with a high degree of certainty in outcomes |
| II | Well-designed comparative studies such as nonrandomized cohorts, case-control studies | Interventions supported can be recommended with a moderate degree of certainty but are not definitive evidence |
| III | Descriptive clinical experience, including case series, case reports, expert consensus | Interventions may be recommended based on accumulated clinical experience |

Adapted from Congress of Neurological Surgeons. Guideline development methodology. [https://www.cns.org/guidelines/guideline-development-methodology.](https://www.cns.org/guidelines/guideline-development-methodology.%20Accessed%20August%2022) Accessed August 22, 2025.

**Supplemental Table 3.** Quantitative summary of penetrating posterior dural venous sinus injuries by sinus group (Figure 3 data)

| **Sinus Group** | **Total Cases, n** | **Isolated, n (%)** | **Dominant Mechanism** | **GCS Reported, n (%)** | **Most Common Interventions** | **Mortality, n (%)** |
| --- | --- | --- | --- | --- | --- | --- |
| Transverse sinus (isolated) | 27 | 27 (100) | MW (92.6%) | 3 (11.1) | Suture, vein graft, Gelfoam, ligation | 3 (11.1) |
| Sigmoid sinus (isolated) | 5 | 5 (100) | MW (80.0%) | 1 (20.0) | Packing, suture, muscle stamp, clip + Gelfoam | 1 (20.0) |
| Torcular Herophili (isolated) | 2 | 2 (100) | MW (100%) | 1 (50.0) | Suture + synthetic graft; NS in 1 | 0 (0) |
| Transverse + sigmoid sinus | 4 | 0 (0) | MW 50%; fracture 50% | 2 (50.0) | Hemorrhage control, ligation, TS clipping | 3 (75.0) |
| Transverse sinus + torcular | 5 | 0 (0) | MW (100%) | 0 (0) | Sutures, Gelfoam, muscle stamps | 0 (0) |
| Total | 43 | 34 (79.1) | MW (88.4%) | 7 (16.3) | — | 7 (16.3) |

Abbreviations: GCS, Glasgow Coma Scale; MW, missile wound; NS, not specified; TS, transverse sinus; SS, sigmoid sinus; TH, torcular Herophili.

**SUPPLEMENTAL REFERENCES**

1.	Cushing H. A Study of a Series of Wounds Involving the Brain and Its Enveloping Structures. *J Brit Surg* (1917) 5(20):558-684. doi: 10.1002/bjs.1800052004.

2.	Meirowsky AM. Wounds of Dural Sinuses. *J Neurosurg* (1953) 10(5):496-514. doi: 10.3171/jns.1953.10.5.0496.

3.	Kapp JP, Gielchinsky I. Management of Combat Wounds of the Dural Venous Sinuses. *Surgery* (1972) 71(6):913-7.

4.	Morentin B, Biritxinaga B. Massive Pulmonary Embolization by Cerebral Tissue after Head Trauma in an Adult Male. *Am J Forensic Med Pathol* (2006) 27(3):268-70. doi: 10.1097/01.paf.0000233550.07598.56.

5.	Paluszkiewicz P, Dudek W, Hart CA. Injury of Left Sigmoid Sinus Treated with Direct Packing and Recombinant Factor Viia: A Case Report. *Injury Extra* (2008) 39(11):364-5. doi: 10.1016/j.injury.2008.07.001.

6.	Pricola KL, Zou H, Chang SD. Successful Repair of a Gunshot Wound to the Head with Retained Bullet in the Torcular Herophili. *World Neurosurg* (2011) 76(3-4):362 e1-4. doi: 10.1016/j.wneu.2010.03.021.

7.	Beaty NB, Diaz C, Crandall K, Sansur C. Penetrating Intracranial Gunshot Wound Transecting the Right Transverse Sinus. *BMJ Case Rep* (2012) 2012:bcr2012006679. Epub 20120917. doi: 10.1136/bcr-2012-006679.

8.	Abdel-Aziz KI, Ragaee MA. Compound Depressed Fracture over Dural Venous Sinuses: Experience of 22 Surgically Treated Cases. *Med J Cairo Univ* (2019) 87(4):2587-91.

9.	Suhendar A, Firdha Huzaima R, Ary Wibowo A. Pediatric Gunshot Penetrating on Transverse Sinus without Graft. *Interdiscip Neurosurg Adv Tech Case Manag* (2022) 27:101427. doi: 10.1016/j.inat.2021.101427.

10.	Patil SS, Devani KK, Lemle SD. Anesthesia Management of a Patient with Penetrating Brain Injury with a Knife into the Occipital Region. *J Neuroanaesthesiol Crit Care* (2024) 11(01):056-60. doi: 10.1055/s-0043-1774302.
